# Production of surfactant-stable keratinase from *Bacillus cereus* YQ15 and its application as detergent additive

**DOI:** 10.1186/s12896-022-00757-3

**Published:** 2022-09-08

**Authors:** Rong-Xian Zhang, Zhong-Wei Wu, Hai-Yang Cui, Ying-Nan Chai, Cheng-Wei Hua, Peng Wang, Lan Li, Tian-You Yang

**Affiliations:** 1 School of Life Science and Technology, Henan Institute of Science and Technology, Xinxiang, 453003 People’s Republic of China; 2 Blood Transfusion Department, The Third Affiliated Hospital of Xinxiang Medical University, Xinxiang, 453003 People’s Republic of China

**Keywords:** Keratinase, *Bacillus cereus*, Production, Detergent compatibility

## Abstract

**Background:**

With the growing concern for the environment, there are trends that bio-utilization of keratinous waste by keratinases could ease the heavy burden of keratinous waste from the poultry processing and leather industry. Especially surfactant-stable keratinases are beneficial for the detergent industry. Therefore, the production of keratinase by *Bacillus cereus* YQ15 was improved; the characterization and use of keratinase in detergent were also studied.

**Results:**

A novel alkaline keratinase-producing bacterium YQ15 was isolated from feather keratin-rich soil and was identified as *Bacillus cereus*. Based on the improvement of medium components and culture conditions, the maximum keratinase activity (925 U/mL) was obtained after 36 h of cultivation under conditions of 35 °C and 160 rpm. Moreover, it was observed that the optimal reacting temperature and pH of the keratinase are 60 °C and 10.0, respectively; the activity was severely inhibited by PMSF and EDTA. On the contrary, the keratinase showed remarkable stability in the existence of the various surfactants, including SDS, Tween 20, Tween 60, Tween 80, and Triton X-100. Especially, 5% of Tween 20 and Tween 60 increased the activity by 100% and 60%, respectively. Furtherly, the keratinase revealed high efficiency in removing blood stains.

**Conclusion:**

The excellent compatibility with commercial detergents and the high washing efficiency of removing blood stains suggested its suitability for potential application as a bio-detergent additive.

**Supplementary Information:**

The online version contains supplementary material available at 10.1186/s12896-022-00757-3.

## Introduction

An important conceptual advance in health and the environment has been recognized that enzymes play a key role in the green processing industries, which are important in improving of the living quality [1]. Keratin is the main structural component of pervasive animal keratinous tissues, such as feathers, wool, claws, etc. Abundant disulfide bonds and hydrogen bonds determine the impressive durability and resistance of keratin structure to common chemical reagents and traditional enzymes, such as pepsin, and trypsin [2]. Microbial keratinase (EC 3.4.21/24/99.11), can specifically decompose recalcitrant keratin protein into soluble peptides or amino acids. The admirable capability of keratinase has vitalized the preference of many experts. Mainly due to its unique keratinous substrate specificity, as well as its high stability and activity at alkaline pH, keratinase has been considered as having industrial potential in a wide variety of fields, including in detergent, dehairing operation in leather, feed industries, and waste management [3–6].

Keratinases are secreted by various microorganisms, including fungi (such as *Aspergillus terreus*) [7], actinomycetes (such as *Streptomyces* sp.) [8], and bacteria especially *Bacillus* species (*B. licheniformis*, *B. pumilus*, *B. subtilus* and *Bacillus* sp.) [9–12]. Of particular interest, alkaline keratinolytic protease was preferred in the detergent used as the cleaning additive to facilitate releasing the proteins, because the laundry detergent pH is generally in the range of 9.0–12.0 [7, 10]. Therefore, to encounter harsh working conditions of detergents, the robustness of keratinase was required, which included the efficiency and stability of enzymes under alkaline condition and the higher temperature, compatibility with surfactants, and healthy dirt removal effects.

Acquisition of novel microorganisms is the basis of the bioprocess advance. A novel keratinase-producing bacterium, *Bacillus cereus* YQ15 from the soil of a poultry housing was isolated and identified. Then, both of media components and physical factors were screened to promote keratinase production by the bacterium, as biosynthesis of keratinase is actively induced by keratinous material and varied greatly with microbial nutritional and conditional requirements [6, 13]. Additionally, because the purification of enzyme costs highly which leads to the preference for crude enzyme rather than purified enzyme during the industrial application, the characterization of crude keratinase was assessed. Interestingly, the keratinase was remarkably stable and the activity was even enhanced in the presence of the various surfactants. The desired compatibility with the commercial detergents was also detected. Furthermore, the performance of its potential in detergent was assessed.

## Materials and methods

### Chemicals and reagents

The molecular test-related materials, such as the DNA marker, pMD-19 T vector, T4 DNA ligase, and Ex taq were obtained from Takara bio (Japan). The soluble keratin substrate (5%) was acquired from J&K Scientific Ltd. (Beijing, China). Type-I collagen, BSA, and DTNB were supplied from Macklin Biochemical Co., Ltd (Shanghai, China). Feather and goat skin were from the local markets. All the other reagents were analytical grade and from commercial sources.

### Strains and media

Feather-dumping soil samples were collected from a chicken ranch in Hui county (Xinxiang, China). The enrichment medium contained: 1% feather meal, 0.1% yeast extract, and salt solution (0.05% NH_4_Cl, 0.05% NaCl, 0.03% K_2_HPO_4_, 0.04% KH_2_PO_4_ and 0.01% MgCl_2_·6H_2_O), pH 7.2. The components of the preliminary screening medium were 0.9% NaCl, 1% skimmed milk powder, and 2.0% agar, neutral pH. The secondary screening medium was composed of 1% feather meal and salt solution, pH 7.2. The basal medium was composed of 1% feather meal, 0.05% NaCl, 0.14% K_2_HPO_4_·3H_2_O, 0.07% KH_2_PO_4_, 0.01% MgCl_2_·6H_2_O, and pH 7.2. LB medium was used as the seed medium for screening and cultivating bacterial strains and recombinant strains.

### Isolation and screening of feather-degrading bacteria

After the soil sample (1 g) was completely dispersed in sterilized saline solution (20 mL), 1 mL of the supernatant was cultivated in the enrichment medium (25 mL) in 250 mL flasks at 37 °C and 200 rpm for 6 h. Then, the serial dilutions of the sample were spread on the slim milk agar plates at 37 °C for 48 h. Colonies with clear zones on slim milk agar were further inoculated on the secondary screening medium using feather keratin as the sole carbon and nitrogen source. After cross-fostering on the preliminary screening medium and the secondary screening medium, keratinase activities of the isolated bacteria were tested to select strains for further study.

### Keratinase determination

Due to the special ability of keratinase, the keratinase activity was tested with keratin as the substrate. Keratinase assay was evaluated using the method described by Zhang et al. [14]. One half milliliter of properly diluted enzyme solution and 1.5 mL 1.0% soluble keratin composed the reaction system. After incubation at 40 °C for 20 min, the reaction was terminated by adding 2 mL of 0.4 M TCA, and maintained at 20 °C for 20 min and then proceed to centrifugation (10,000 × *g*, 10 min) with centrifugate (Xiangyi, H1650-W, China). Folin-phenol chromogenic reaction was performed at 40 °C for 20 min by adding the following components in sequential order: 0.5 mL of the above supernatant, 2.5 mL Na_2_CO_3_ solution (0.4 M), and 0.5 mL Bradford reagent. One unit of keratinase activity (1 U) was defined as the rise of 0.01 absorbance at the wavelength of 660 nm measured under the reaction conditions.

### Identification of the bacterial strain

#### Physiological and biochemical identification

Physio-biochemical characteristics of the strain YQ15 were identified by consulting with Bergey’s manual of systematic Bacteriology [15]. TEM (Hitachi HT7700, Japan) was adopted to take images of the strain.

#### Molecular identification

The molecular identification was performed according to the method described by Zhang et al. [14]. The target 16S rDNA fragment was sequenced by GeneCreate Biological Engineering Co., Ltd. (Wuhan, China). After blast in NCBI (https://www.ncbi.nlm.nih.gov/), the phylogenetic tree was constructed by aligning the 16S rDNA sequence of the strain YQ15 and the related reported sequences by using the neighbor-joining method by the MEGA 7 software.

### Seed preparation

The strain YQ15 from frozen glycerol stock was activated by being cultivated on seed-agar medium at 37 °C for 24 h. Then the produced culture was inoculated into the seed medium for preparing the inoculum. Subsequently, the fresh culture was inoculated into the fermentation medium for keratinase production.

### Promotion of keratinase production

The influence of media components and fermentation conditions for keratinase production by the strain YQ15 were investigated using the one-factor-at-a-time method. The evaluation was carried out including not only media components, such as keratinase inducers, carbon sources, nitrogen sources, and metal ions, but also cultivation conditions, such as initial pH, temperature, rotatory speed, and inoculum size. Importantly, because the insolubility of medium components disturbed the exact test of the biomass of the strain, the production of keratinase by the strain YQ15 in the fermentation process was considered as the major evaluation parameter.

### Keratinase preparation

After fermentation, the supernatant was collected by centrifugation with centrifugate (Xiangyi, H1850R, China) at 4746 × *g* and 4 °C for 15 min. And the supernatant containing keratinase was used for further analysis.

### Characterization of keratinase

#### Effects of pH and temperature on the keratinase activity

The optima pH was detected in pH buffer at 50 mM from pH 5.0–12.0 (citrate phosphate-sodium citrate, pH 5.0–6.0; sodium phosphate, pH 7.0–8.0; glycine–NaOH, 9.0–10.0; disodium hydrogen phosphate-NaOH, 11.0–12.0). The optimal temperature was measured at the temperature range of 20–80 °C at intervals of 10 °C, with 1% soluble keratin as the substrate. And pH stability of the keratinase was also assayed at the corresponding pH after incubating at room temperature for 1 h. And thermal-stability of the keratinase was assayed by keeping the enzyme for 1 h at the corresponding temperatures.

#### Effect of chemicals

The effects of various chemicals, including metal ions, inhibitors, reducing agents, and surfactants on keratinase activity were evaluated by premixing the enzyme with the respective chemical reagents at the different concentrations at room temperature for 30 min. The residual activities were determined at pH 9.0 (50 mM glycine–NaOH buffer) and 60 °C. The control was assayed in absence of any chemicals under the same reacting conditions.

The effects of the typical inhibitors and reducing agents at the concentration of 5 mM on the keratinase activity were determined, including PMSF, EDTA, and *β*-ME. The surfactants, ionic and nonionic surfactants, such as Tween 20, Tween 60, Tween 80, Triton X-100, and SDS, were used to determine their influences on the keratinase activity. The activities of keratinase were assayed under the above conditions.

#### Compatibility of the *B. cereus* keratinase with commercial detergents

A variety of famous brands of commercial detergents at the concentrations of 0.7 and 1.0 mg/mL were used to test the compatibility of the keratinase with commercial detergents because the used concentrations of detergent in laundry are less than 0.9% (w/v) in cleaning laundry.

Pre-processing inactivation of endogenous enzymes in detergents was conducted by boiling the detergents for 30 min. The residual activity was determined after incubation of the enzyme solution with the various detergents at different concentrations at 50 °C for 30 min. The treatment in absence of detergent was taken as 100%.

#### Substrate specificity

To further explore the potential application of YQ15 keratinase, substrate specificity was assayed with various substrates including keratinous substrates (human hair, chicken feather, wool powder, keratin), and protein substrates (azocasein, casein, and type-I collagen), according to the methods described by Zhang et al. [16]. The reaction of the enzyme toward the insoluble substrates is that the reaction mixtures including 0.5 g substrate in the pH of 9.0 buffer (50 mM glycine-NaOH) and 0.5 mL proper diluted enzyme solution were maintained at 60 °C for 60 min, the reaction was stopped by adding the TCA solution (0.4 M, 1 mL), and the mixture’s supernatant was collected after centrifugation at 10000 × g for 10 min, then the absorbance at 280 nm was measured for human hair, chicken feather powder, and wool powder. And the methods used for detecting enzyme activities on type-I collagen substrate, BSA, and casein were the same as the keratinase assay described above.

### Evaluation of washing efficiency

Because of its amazing compatibility with detergents, the keratinase exhibited the potential as an additive in detergent formulas. The evaluation of its washing efficiency was carried out by removing the blood stains from the cotton cloth (9 cm × 9 cm) at the conditions of laundry. The blood-stained cotton cloth pieces were obtained with the procedures by Zhang et al. [17]. Then, the cloth pieces were treated as the following: a, negative control: 100 mL tap water at 25 °C for 20 min; b, positive control: 100 mL of 0.7% commercial detergent at 25 °C for 20 min; c, 100 mL of crude enzyme solution (400 U/mL); d, target crude enzyme (200 U/mL) with 0.7% inactive commercial detergent in tap water for 20 min. After rinsing and drying, visual examination of different treatments with cloth pieces was performed. All the treated samples were incubated at 60 °C. The washing efficiencies of blood stains were assessed by the camera and naked eyes.

### Statistical analysis

Assays were carried out in triplicate. The data were statistically analyzed with the SAS 9.4 software and were shown as the mean of three replicates ± standard deviation (mean ± SD).

## Results and discussion

### Isolation and identification of feather-degrading strain

To explore the microorganism resource for degrading feather waste, the keratinase-producing strains were screened using feather powder as the sole carbon, nitrogen source from the feather dumped soil sample. It was observed that fifty-three keratinase-producing bacterial strains were newly isolated. According to the ratios of transparent zones’ diameters to those of colonies on slim milk plates combining the keratinase assay, fifteen stains were selected with high ability of keratinase production. The strain YQ15 showed clear zone on skimmed milk plate and also displayed high activity towards feather keratinous substrate and commercial keratin substrate, and was thus selected for further research.

**Fig. 1:**
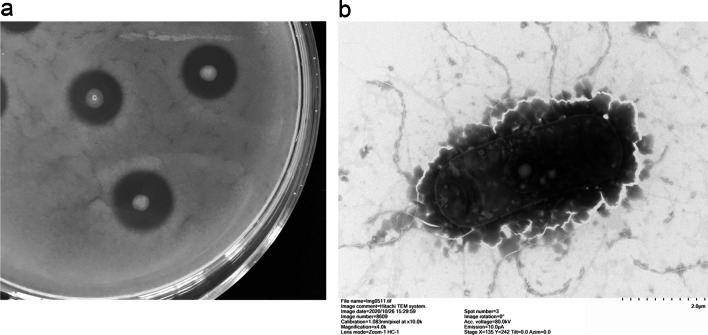
Morphological graphs of *B. cereus* YQ15. **a**, the colony on skimmed milk agar medium; **b**, the bacterial image taken by TEM

**Fig. 2:**
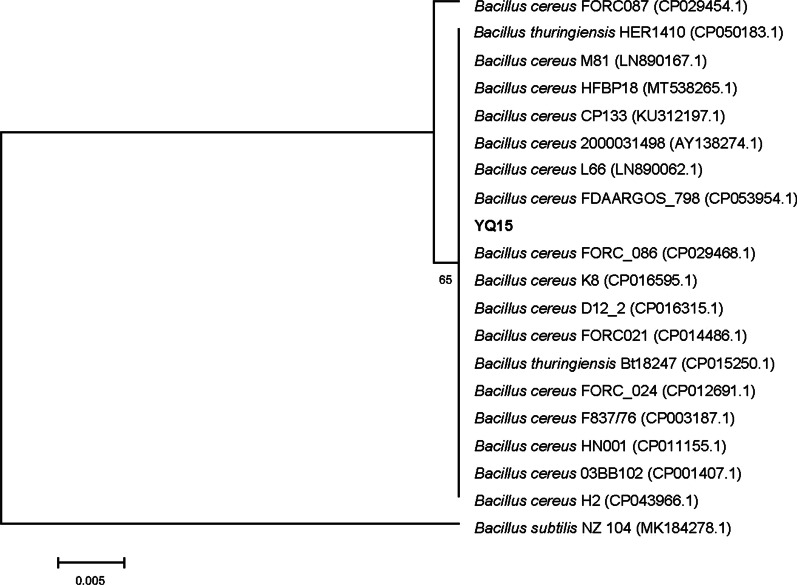
Neighbor-joining phylogenetic tree of the strain YQ15 based on the 16S rDNA gene sequences (Bootstrap values were based on 1000 replicates). The bar represents 0.005 substitutions per site. The 16S rDNA sequence of *B. subtilis* NZ 104 (MK184278) was used as the outgroup. The related bacterial accession numbers from NCBI database were showed in parentheses

The colony of YQ15 has an irregular edge and a rough surface with grayish-white color, after cultivation at 37 °C for 24 h on LB agar plate. No bacteria growth was observed when the culturing temperatures exceeded 50 °C. And the micromorphology of the strain was Gram-positive and rod-shaped (3–5 μm by 0.5–1 μm) (Fig. 1). The above results showed the isolate belonged to the *Bacillus* genus. To further identify the isolate, a 1518 bp fragment of the 16S rDNA sequence was deposited to GenBank database (Accession number: MW282806). After the blast in the NCBI database, the related sequences were downloaded. The results of alignment and phylogenetic analysis indicated that the strain YQ15 belongs to *B. cereus* (CP053954, CP029468, LN890062) with 99% similarity. It could be observed from the phylogenetic tree constructed based on 16S rDNA sequences that the strain was in the clade of *B. cereus* (Fig. 2) which belongs to the most promising keratinase producer species. Briefly, the results verified that the isolate should be designated *B. cereus* YQ15.

### Time profiles of growth and keratinase production

The studies on the growth and enzyme production of YQ15 were carried out under time intervals of 4 h up to 40 and 68 h, respectively. Time profiles of growth and keratinase production of YQ15 were shown in more detail (see Additional file 1). The growth of YQ15 in the seed medium was low during the first 4 h, but reached the maximal strain concentration at 16 h during the 12–20 h, and entered the decline stage after 20 h. Therefore, the optimal culture time of seed culture was 16 h. The time course of keratinase production by YQ15 showed that the enzyme began to be produced from 4 h, then, increased rapidly from 8 to 36 h. The peak of keratinase yield (220 U/mL) was obtained after 36 h cultivation. Thereafter, the keratinase decreased to 50 U/mL. Hence, the optimal production of keratinase by *B. cereus* YQ15 was 36 h. A similar trend has been reported by Nilegaonkar for *B. cereus* MCM B-326 [18].

### Improvement production of keratinolytic enzyme

Production of keratinase by *Bacillus* species has been reported [13, 19]. And it is well known that the production of keratinase by microorganisms is actively induced by keratinous material, and varies with microbial nutritional and conditional requirements. Therefore, the effects of medium components and physical factors on the bacterial keratinase production have been investigated.

#### Effect of medium composition

**Fig. 3:**
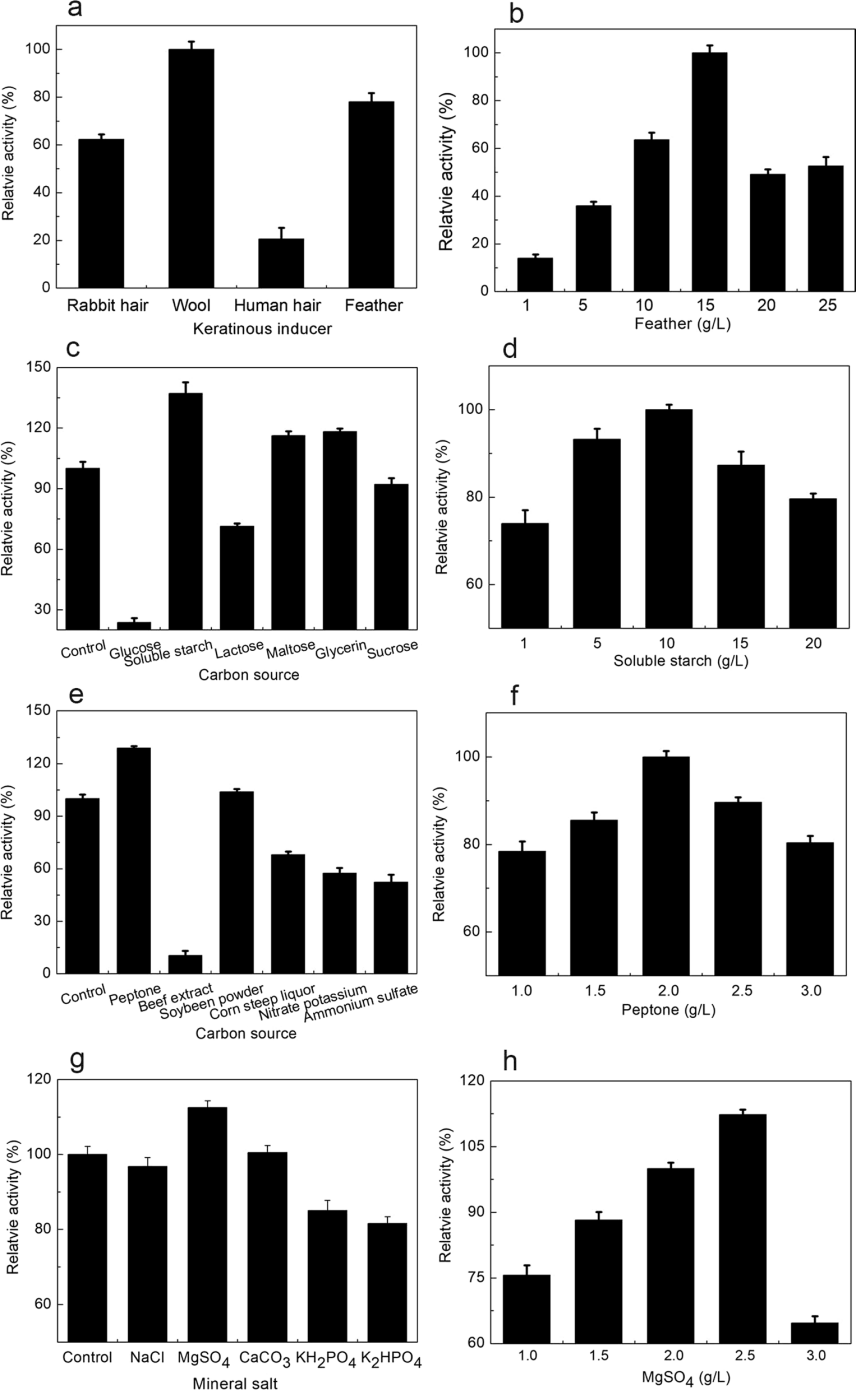
Effects of medium components on keratinase production by *B. cereus* YQ15 with an initial pH of 7.2 after cultivation at 37 °C and 200 rpm for 36 h. **a**, Keratinous inducers (10 g/L); **b**, Feather; **c**, Carbon sources (10 g/L); **d**, Soluble starch; **e**, Nitrogen sources (2 g/L); **f**, Peptone; **g**, Mineral salts (2 g/L); **h**, MgSO_4_

Microbial keratinase production is usually stimulated by keratinous induction [20, 21]. Several typical keratinous inducers (such as feather, hair, and wool) thus were used. According to the results, wool keratin and feather keratin powder were the effective inducers on production of keratinase by *B. cereus* YQ15 (Fig. 3a). Given the great amount of chicken feather yielded annually, feather was selected for further study. Based on the results, the amount of keratinase production varied with the concentration of feather meal in the medium. The maximal keratinase activity (220 U/mL) was obtained when chicken feather meal was 15 g/L (Fig. 3b). There have also been several reports on chicken feather enhancing the keratinase production by some bacteria at a concentration range of 1.0 to 10.0 g/L [22]. However, it is further discovered that the complete feather could not be digested obviously by *B. cereus* YQ15 keratinase. It is similar to the enzymes from *B. cereus* IIPK36 and *Bacillus* sp. WF146 [23, 24]. We speculated that the reason for it might be that those enzymes lack disulfide bond-reducing potential, which cleaves the disulfide bond to accelerate decomposing of feather’s recalcitrant structure [4].

Besides keratin materials, the carbon source, nitrogen source, and minerals, supplemented in the medium, are likely to improve the extracellular keratinase production. Subsequently, the effects of supplemented carbon source, nitrogen source, and minerals on extracellular keratinase biosynthesized by *B. cereus* YQ15 were also studied by using only factor analysis of variance. The findings showed that keratinase production varied with those tested sources (Fig. 3c–f).

As the type of microbial inducible enzyme, available carbon source, such as glucose, is not good for microorganisms to produce keratinase. Compared to the control, the supplemented soluble starch, maltose, and glycerin showed the activating effects on keratinase production by *B. cereus* YQ15, among the tested carbon sources, which were 137.21%, 116.21 and 118.24%, respectively. And the maximal keratinase (303 U/mL) was detected when the medium was supplemented with 10 g/L of soluble starch (Fig. 3d). It is consistent with the enhancing effects on the keratinases production by *B. pseudofirmus* FA30-01 and *Streptomyces gulbargensis* [25, 26]. Moreover, several nitrogen sources were studied for the keratinase production. And the results showed that peptone was the most favorable nitrogen source for keratinase production by *B. cereus* YQ15 (Fig. 3e, f). The highest activity of keratinase (390 U/mL) was obtained when the peptone was at the concentration of 2.0 g/L. Furtherly, the influence of various minerals in the medium was studied for keratinase production by *B. cereus* YQ15. According to the results, magnesium ion has an activating influence on keratinase biosynthesis among the tested minerals (Fig. 3g, h). And the keratinase activity of 493 U/mL was detected in the presence of magnesium sulfate at the concentration of 2.5 g/L. It was also reported by Fakhfakh-Zouari et al. that magnesium ions increased the keratinase production by *B. pumilus* A1 [27].

In conclusion, the optimal medium composition is 1.5% feather meal, 1% soluble starch, 0.2% peptone, and 0.25% MgSO_4_, 0.05% NaCl, 0.14% K_2_HPO_4_·3H_2_O, 0.07% KH_2_PO_4_, 0.01% MgCl_2_·6H_2_O, and pH 7.2. The enzyme activity of 493 U/mL was obtained with the optimum medium after cultivation at 37 °C and 200 rpm for 36 h.

#### Effect of culture conditions on keratinase production

**Fig. 4:**
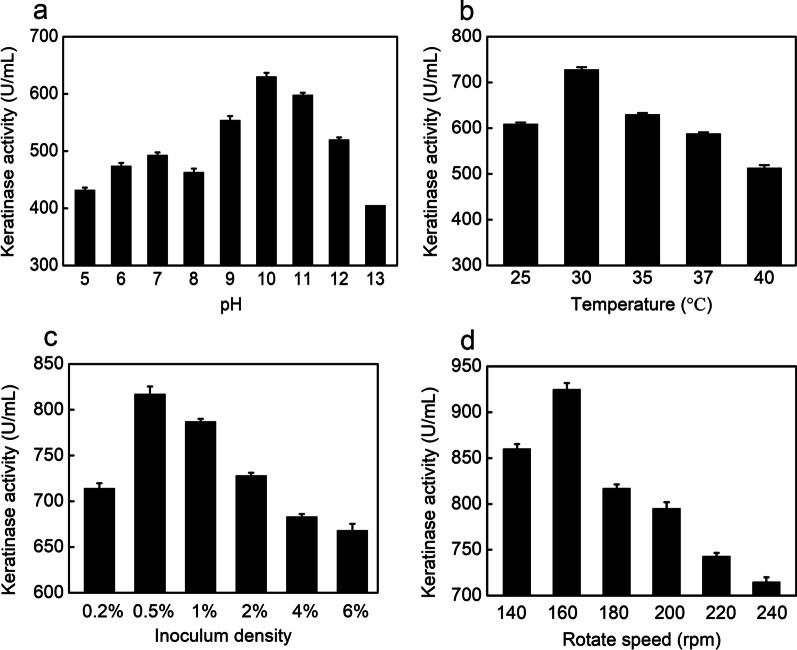
Effect of culturing conditions on the keratinase production by *B. cereus* YQ15 using the optimal medium. **a**, pH; **b**, Temperature; **c**, Inoculum density; **d**, Rotate speed

The proper condition is beneficial for bacterial growth, and production of extracellular enzymes via affecting absorbance and utilization of nutrients by microorganisms and interfering activities of enzymes in microbial metabolic pathways. Furtherly, the initial pH of the medium affected microbial production of keratinase. The pH effects on keratinase production by *B. cereus* YQ15 were also studied. The results showed that when the medium pH is in the range of 5.0 to 12.0, *B. cereus* YQ15 could produce keratinase, and high keratinase activities (more than 500 U/mL) were detected in the range of pH 9.0 to 11.0. The optimum initial pH of the medium for keratinase production was pH 10.0 (Fig. 4a)**.** Beyond that range, the keratinase activity decreased dramatically. It is accordant with that the general bacteria prefer an alkaline pH range for enzyme production [12, 28]. Medium pH is important for microorganisms to grow and produce the target in several ways. Firstly, it affects the activities of various intracellular enzymes, leading to changes in microbial metabolic pathways. Secondly, pH also affects the morphology and membrane permeability of microorganisms, thus influencing microbial absorption of nutrients components from the culture medium and the secretion of metabolites.

The optimum for keratinase production occurred with an inoculum size of 0.5% (v/v) when incubating at 35 °C and 160 rpm (Fig. 4b–d). The optimal inoculum size differed from that of *B. subtilis* KD-N2 (10%) [29]. And our finding is similar to *B. cereus* IIPK35, whose inoculum size was 2% for high keratinase production [23]. Fermenting temperature usually influences the metabolism of microorganisms and exhibits a significant impact on the biosynthesis of target enzymes. In this study, the optimum growth temperature for keratinase production is 35 °C, which is similar to some other bacterial keratinases with an optimum temperature range of 30 to 40 °C [23, 30, 31]. The results indicated that *B. cereus* YQ15 belongs to mesophilic bacteria, exhibiting superior potential in industrial application than the thermophiles because of the less energy cost from this perspective. Oxygen transfer has been reported as a vital factor for microbial enzyme synthesis [32]. Due to the low solubility of oxygen in the fermentation broth, continuous stirring is required to meet the oxygen demand of aerobic microorganisms. Also, considering the low viscosity of the medium, moderate dissolved oxygen is beneficial to bacterial growth, accumulation of biomass, and biosynthesis of products. But too much dissolved oxygen can inhibit the synthesis of products and waste energy. The findings are that the proper rotation speed (160 rpm) ensures the oxygen transfer efficiency for *B. cereus* YQ15 (Fig. 4d). The attributes of *B. cereus* YQ15 are energy-saving and economically attractive for producing keratinase.

Based on the optimal medium composition and optimal cultivating conditions, the keratinase yield (925 U/mL) was obtained after 36 h cultivation. Thereafter, the keratinase decreased. The high productive efficiency of keratinase produced by *B. cereus* YQ15 indicated its potential use in the biotechnological industry. The results also suggested that keratinase production by *B. cereus* YQ15 was related to its primary metabolism, which is similar to the production of keratinases by *B. pumilus* GRK and *Chryseobacterium* sp. Kr6 [31]. Summarily, keratinase production was enhanced by 4.2-fold after the improvement procedures, compared to the initial keratinase yield.

### Characterization of YQ15 keratinase

#### Effects of pH and temperature

**Fig. 5:**
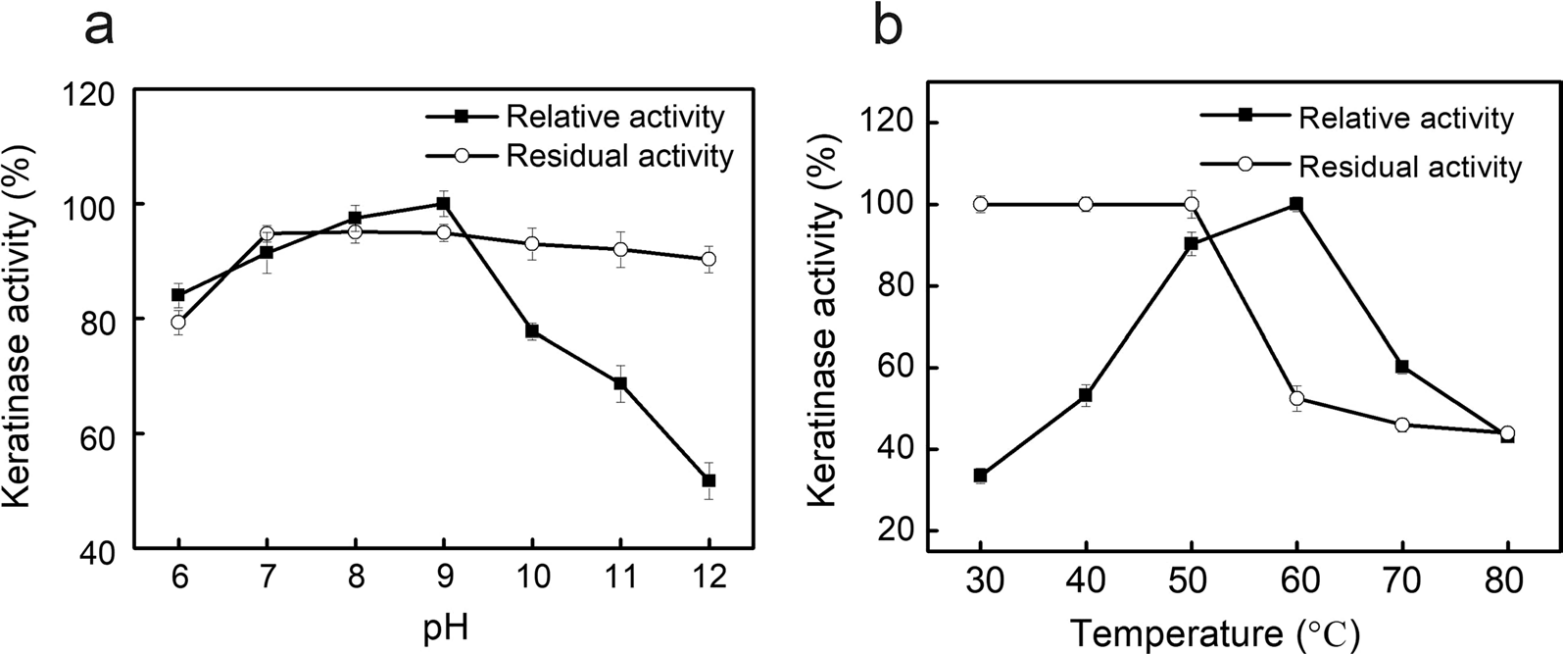
Effects of pH and temperature on the keratinase activity. **a**, the optimal pH and pH stability of keratinase; **b**, the optimal temperature and thermal stability of keratinase. Black square and hollow circle indicated the enzyme activity and stability, respectively

pH usually affects the activity and stability of enzymes by means of changing not only the protonation or deprotonation states of side chain groups of protein conformation, but also the conformation of substrate. As shown in Fig. 5a, the keratinase has the optimal pH at 9.0 and exhibited moderated activity at the pH range (6.0–9.0). Many bacterial keratinases have been reported with optimal pH at pH 9.0 [27, 33, 34]. The reason might be that alkaline condition is not only beneficial for stabilizing the active conformation of enzyme’s three-dimensional structure but also for unfolding the structure of keratin. Also, the results showed that the keratinase had attractive pH stability in the wide pH range of 7.0–12.0, more than 90% of the residual activities were retained after the treatment. It is reported that proteases applied as an additive in detergent formulation are required to be stable at a pH range of 8.0–10.0 [5, 10]. Therefore, the remarkable pH stability of *B. cereus* YQ15 encouraged its potential use in detergents, which is similar to the findings of the keratinases produced by *Paenibacillus woosongensis* TKB2 and *Bacillus* sp. NDS-10 [35, 36].

Optimal reacting temperature of the enzyme was 60 °C. When the temperature exceeds 60℃, the relative activity decreases obviously (Fig. 5b). Some keratinases of mesophilic bacteria, such as *Brevibacillus parabrevis*, *B. licheniformis* RP1 and *B. subtilis* FTC02PR1, also have the optimal temperature of 60 °C [10, 14, 37]. And the thermal-stability analysis suggested that keratinase exhibited extremely stable in the temperature range of 30–50 °C, and high temperature lead to loss of keratinase activity. The thermal stability indicated that the keratinase performed as a good detergent enzyme [5]. The findings are accordant with keratinases isolated from mesophilic microorganisms, such as *B. pumilus* GRK, *B. licheniformis* N22, and *B. subtilis* LFB-FIOCRUZ 1266, which exhibited high thermal stability in the range of 30–45 °C [22, 31, 38].

#### Effects of various chemicals

According to the sensitivity to inhibitors, proteases could be classified. As shown in Table 1, *B. cereus* YQ15 keratinase suffered severely inhibiting influence in the presence of PMSF and EDTA, the famous inhibitors of serine protease and metallo-protease. The results suggest that the *B. cereus* YQ15 keratinase belongs to serine and metallo-proteases, indicating the active conformation of the enzyme requires the presence of metal cofactor. The findings are accordant with the keratinases produced by *Br. parabrevis* CGMCC 10,798, *B. pumilus* A1, and *B. pumilus* NRC21 [14, 39, 40]. Moreover, no obvious influence on keratinase activity was observed in the presence of *β*-ME. Furtherly, it is excited that the keratinase was remarkably stable against various surfactants (SDS, Tween 20, Tween 60, Tween 80, and Triton X-100), and even stimulated by 5% of Tween 20 and Tween 60 with the increase of 1.0-fold and 0.6-fold, respectively. The results indicated the attractive advantage of *B. cereus* YQ15 keratinase in promising application in the detergent industry. Similarly, the *Brevibacillus* sp. AS-S10-II keratinase has been reported as a good candidate for detergent formulation [32]. The excellent stability of enzymes with various surfactants for industrial purposes is the distinctive advantage of promising application because surfactants are common ingredients in detergents for improving their washing efficiency [5, 32].

**Table 1:** Effects of protease inhibitors, reducing reagent, and surfactants on the keratinase activity

Chemicals	Concentration	Relative activity (%)
PMSF	5 mM	ND*
EDTA	5 mM	6.17 ± 0.52
*β*-ME	5 mM	97.53 ± 4.31
SDS	1%	101.91 ± 2.63^c^
Tween 20	5%	200.26 ± 7.35^a^
Tween 60	5%	163.62 ± 8.23^b^
Tween 80	5%	108.98 ± 2.26^c^
Triton X-100	5%	107.51 ± 7.13^c^

*ND means that no activity was detected

Different letters represent a value significantly greater than the control value

Because activity of metallo-protease varies with the presence of metal ions, the influence of metal ions on the keratinase activity was accordingly studied. As shown in Table 2, it is observed that the metal ions of Zn^2+^, Mn^2+^, Na^+^, Co^2+^, Ca^2+^, and Mg^2+^ showed significant stimulatory effects on keratinase activity. These metal ions might contribute to the protection of the enzyme’s configuration from unfolding, and to the promotion of generating covalent transition products between substrate molecular and enzyme molecular. Especially, the presence of Mn^2+^ at 10 mM enhanced enzyme activity by more than 1.8 folds, which is accordant with the keratinase produced by *B. thuringiensi*s AD-12 [41]. Interestingly, Co^2+^ was observed stimulatory effect on keratinase activity, which has been also reported by Jaouadi et al. on *Brevibacillus brevis* US575 keratinase [42]. Moreover, Ca^2+^, Mg^2+^, and Zn^2+^ enhanced obviously keratinase activity. The stimulating effects on various keratinase have been reported [42, 43]. It is speculated that metal ions are responsible for the forming of ion bridge or salt bridge of protein structure and the distribution of water molecules on the surface of protein structure. Thus, the hydrophobicity of inner core and conformation stability of protein structure were further promoted [12, 44]. On the contrary, the *B. cereus* YQ15 keratinase activity was inhibited severely by Fe^2+^ and Al^3+^, even though no activity was detected in the presence of Cu^2+^, Pb^2+^, and Fe^3+^.

**Table 2:** Effects of variable metal ions on the keratinase activity

Metal ions	Relative activity (%) at 10 mM
Zn^2+^	160.39 ± 1.84^c^
Mn^2+^	287.86 ± 4.88^a^
Na^+^	120.48 ± 0.07^e^
Co^2+^	132.74 ± 2.47^d^
Fe^3+^	ND
Ca^2+^	170.61 ± 1.13^b^
Mg^2+^	160.70 ± 0.92^c^
K^+^	98.08 ± 0.49
Al^3+^	38.14 ± 1.34
Cu^2+^	ND
Pb^2+^	ND
Fe^2+^	57.28 ± 0.64

ND means that no activity was detected

Different letters represent a value significantly greater than the control value

#### Compatibility with commercial detergents

Commercial laundry detergents usually contain oxidants, surfactants, bleaching agents, and softening builders, which can influence the enzyme’s catalytic efficiency and stability [32]. Therefore, a good detergent protease performs well in the presence of those chemicals. Regarding the excellent stability and even activation effects of the keratinase in presence of various surfactants, the compatibility of the keratinase with commercial detergents was also evaluated to test its potential as a detergent additive.

**Fig. 6:**
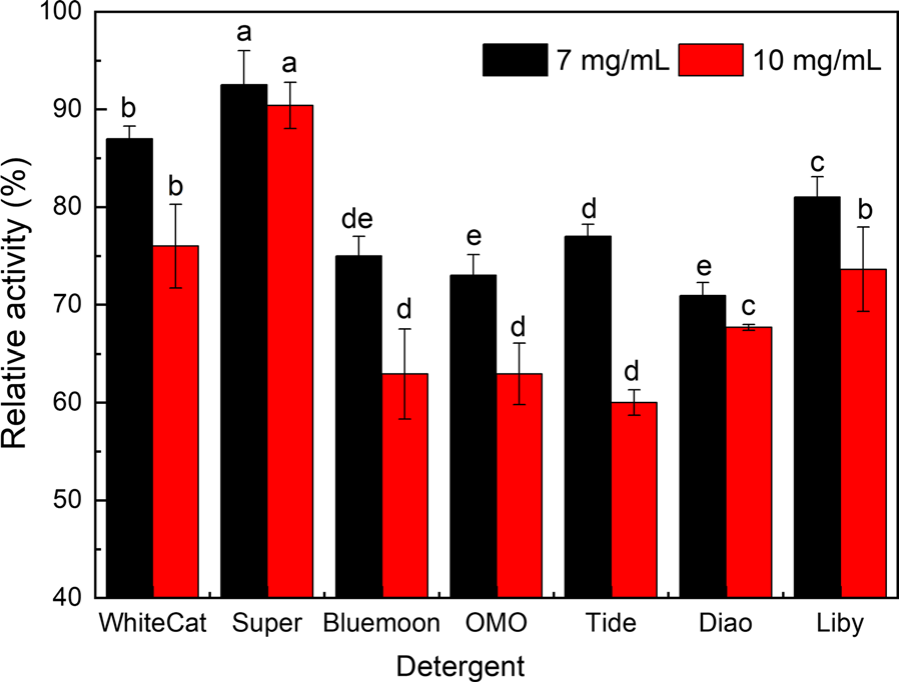
Compatibility and stability of the *B. cereus* YQ15 keratinase with commercial detergents after incubation at 50 °C for 30 min. The keratinase activity in absence of detergent was set as 100%. Different letters at the same concentration indicate significant differences (p ≤ 0.05)

The results showed that *B. cereus* YQ15 keratinase exhibited excellent compatibility and stability with the commercial detergents (Fig. 6). Comparing the control, the residual activities retained more than 70% at 7 mg/mL and more than 60% at the 10 mg/mL in the presence of the tested commercial detergents. It is generally known that the high stability of enzymes with detergents indicated its suitability for use as a detergent additive [23, 45]. Correspondingly, there have been various reports on the compatibility of microbial keratinases with commercial detergents which validated the suitable potential of alkaline keratinases as a detergent additive [33].

#### Substrate specificity

**Fig. 7:**
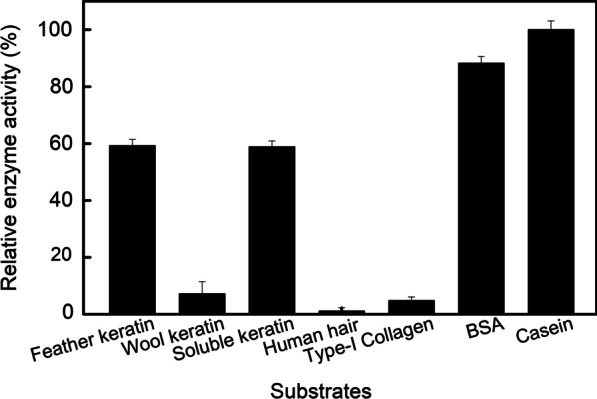
Substrate specificity of the *B. cereus* YQ15 keratinase

The enzyme’s ability to degrade various substrates was detected towards both protein and keratin substrates (Fig. 7). The results showed that the enzyme has a high catalytic capability toward casein and BSA, subsequently, soluble keratin and feather keratin substrates. Additionally, the enzyme exhibited low activity toward type-I collagen substrate, as well as wool keratin and human hair substrate. Furthermore, the high activity toward casein and keratinous substrates, and the low activity on type-I collagen endowed that the keratinase might be potential in detergent industry.

### Washing performance

**Fig. 8:**
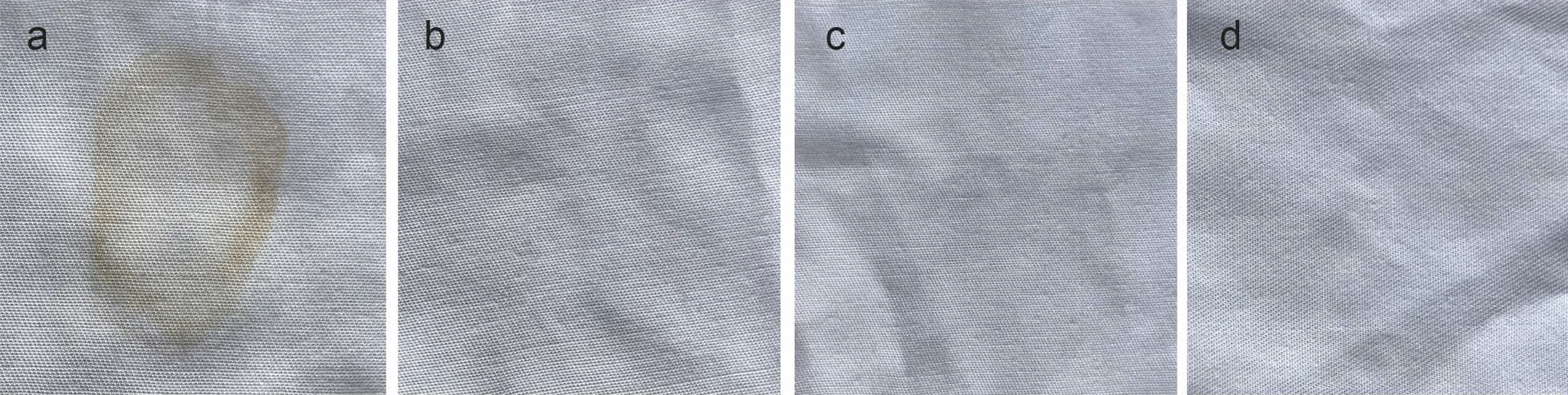
Washing performance of *B. cereus* keratinase. **a**, the stain treated with 100 mL tap water; **b**, the stain treated with 100 mL detergent solution (0.7 mg/mL) for 20 min; **c**, the stain treated with 100 mL of crude enzyme solution (400 U/mL); **d**, the stain treated with 100 mL detergent and enzyme solution (0.7 mg/mL inactive commercial detergent combining with 200 U/mL keratinase) for 20 min

Surfactant-stable keratinases are attractive enzymes as an additive in detergent preparations, as they exhibit high hydrolysis capacity towards various substrates, including keratin. To that end, washing performance of the keratinase was furtherly carried out on removal of blood stains from cotton cloth. The results showed that not only the crude *B. cereus* YQ15 keratinase combined with the inactivated detergents at general washing concentration in tap solution but also the independent crude keratinase, could remove the blood stains after washing within 20 min (Fig. 8). In detail, the stained cloth after the combined treatment has the similar removal efficiency with the detergent treatment. And, no substantial difference was observed between the treatments and positive control, which also indicated there is no damage to cloth fiber after the enzymatic washing. Correspondingly, there have been reported that blood stains on cotton cloth could be removed by various alkaline proteases. For example, the crude keratinase from *B. pumilus* GRK combined with detergent at 7 mg/mL showed removed blood stains at 60 °C, the crude keratinase from *P. woosongenis* TKB2 did it at 50 °C [31, 33]. Additionally, the *B. aerius* NSMk2 keratinase and the *B. safenisis* LAU 13 keratinase removed blood stains after incubation at 30 °C for 30 min and 3 h, respectively [34, 46].

In current, detergent-stable enzymes are popular with consumers with the increasing environmental concern and demand for high efficiency and quality of life. Accordingly, based on the compatibility of the enzyme with the detergent ingredients, the enzyme maintaining efficient functioning is required in industrial utility in the presence of various surfactants. Conclusively, the above results reflect the high washing efficiency of the keratinase, indicating its promising future potential in the laundry industry.

## Conclusion

In this article, a keratinase-producing *B. cereus* YQ15 was isolated from feather keratin-rich soil and was able to grow on the medium using feather keratin as the sole carbon and nitrogen source. Also, the keratinase production was optimized. Moreover, the keratinase exhibited remarkable stability in presence of the various surfactants and even was stimulated by 5% of Tween 20 and Tween 60. Furtherly, the excellent washing efficiency of removing blood stains suggests its suitability for potential application as a bio-detergent additive.

## Supplementary Information

**Additional file 1.** Time profiles of growth and keratinase production.

## Data Availability

The results of the datasets analyzed during the current study were included in the manuscript and the nucleotide sequence of 16S rDNA of *Bacillus cereus* YQ15 was submitted to the NCBI (https://blast.ncbi.nlm.nih.gov) under the accession number MW282806. Any additional information used and analyzed for the current study is available from the corresponding author on reasonable request.

## Abbreviations

BSA: Bovine serum albumin
DTNB: 5, 5’-Dithio bis-(2-nitrobenzoic acid)
LB: Luria Bertani
TCA: Trichloroacetic acid
TEM: Transmission Electron Microscope
NCBI: The National Center of Biotechnology Information
PMSF: Phenylmethanesulfonyl fluoride
EDTA: Ethylenediamine tetraacetic acid
*β*-ME: *β*-Mercaptoethanol
SDS: Sodium dodecyl sulphate
SDS-PAGE: Sodium dodecyl sulfate polyacrylamide gel electrophoresis
R-250: Coomassie Brilliant Blue R-250
BSA: Bull serum albumin
