# Supplementary material for: Production of surfactant-stable keratinase from Bacillus cereus YQ15 and its application as detergent additive
Source: BMC Biotechnol. 2022 Sep 8;22:26. doi: 10.1186/s12896-022-00757-3 (PMC9454225; doi:10.1186/s12896-022-00757-3)
Supplement: Supplementary file 1 — Additional file 1. Time profiles of growth and keratinase production. [file 12896_2022_757_MOESM1_ESM.pdf]

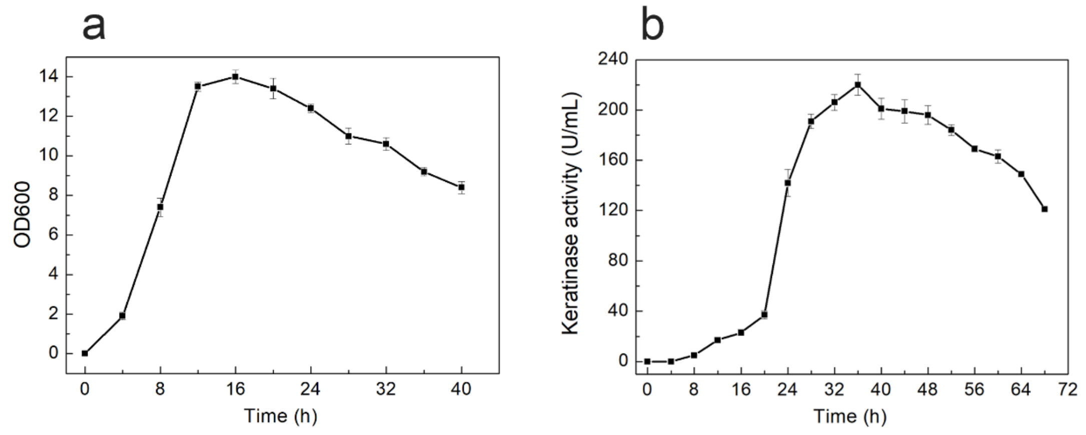

Additional file 1. Time profiles of growth and keratinase production. a, growth curve of *B. cereus* YQ15 in seed medium; b, Time course of keratinase production by *B. cereus* YQ15 in the basal medium.
